# Age-stratified clinical performance and survival of patients with IDH-wildtype glioblastoma homogeneously treated by radiotherapy with concomitant and maintenance temozolomide

**DOI:** 10.1007/s00432-020-03334-3

**Published:** 2020-08-03

**Authors:** Kerstin Berger, Bernd Turowski, Jörg Felsberg, Bastian Malzkorn, Guido Reifenberger, Hans-Jakob Steiger, Wilfried Budach, Jan Haussmann, Johannes Knipps, Marion Rapp, Daniel Hänggi, Michael Sabel, Hendrik-Jan Mijderwijk, Marcel A. Kamp

**Affiliations:** 1 Department of Neurosurgery, Medical Faculty, Heinrich Heine University, Moorenstraße 5, 40225 Düsseldorf, Germany; 2 Institute for Diagnostic and Interventional Radiology, Frankfurt, Germany; 3 Institute for Neuropathology, Freiburg, Germany; 4 Department of Radiation Oncology, Medical Faculty, Heinrich Heine University, Moorenstraße 5, 40225 Düsseldorf, Germany

**Keywords:** Age at diagnosis, Glioblastoma, *MGMT* promoter methylation, Chemoradiotherapy, Survival

## Abstract

**Objective:**

Isocitrate dehydrogenase (IDH)-wildtype glioblastomas are the most malignant glial tumours. Median survival is only 14–16 months after diagnosis, with patients aged ≥ 65 years reportedly showing worse outcome. This study aimed to further evaluate the prognostic role of age in a homogenously treated patient cohort.

**Methods:**

The study includes 132 IDH-wildtype glioblastoma patients treated between 2013 and 2017 with open resection followed by radiotherapy with concomitant and maintenance temozolomide. Patients were dichotomized into a non-elderly (< 65 years) and an elderly (≥ 65 years) group. Extent of resection and the O6-methylguanine-DNA methyltransferase (*MGMT*) promoter methylation status were determined for each tumour. Clinical and radiological follow-up data were obtained at 6 weeks after the end of radiation therapy and thereafter in 3-month intervals. Progression-free survival (PFS) and overall survival (OS) were evaluated in univariate and multivariate cox regression analyses.

**Results:**

The elderly group consisted of 58 patients (median age: 70.5 years) and the non-elderly group of 74 patients (median age: 55 years). Median pre- and postoperative operative Karnofsky Performance Scale (KPS), Eastern Cooperative Oncology Group (ECOG) score and National Institutes of Stroke Scale (NIHSS) were not significantly different between the groups, but KPS and ECOG scores became significantly worse in the elderly group at 6 weeks after termination of radiation therapy. Neither PFS nor OS differed significantly between the age groups. Patients with *MGMT* promoter-methylated tumours survived longer.

**Conclusion:**

Elderly patients in good pre- and postoperative clinical conditions may show similar outcome as younger patients when treated according to standard of care. However, elderly patients may suffer more frequently from clinical deterioration following chemoradiotherapy. In both age groups, *MGMT* promoter methylation was linked to longer PFS and OS.

## Introduction

Isocitrate dehydrogenase (IDH)-wildtype glioblastomas are the most frequent and most malignant intrinsic brain tumours that lead to an average survival of only three months without treatment (Malmstrom A et al. 2012; Tamimi and Juweid 2017). The incidence of IDH-wildtype glioblastoma increases with patient age and peaks between 65 and 75 years of age (Brodbelt et al. (2015); Thakkar et al. 2014). More than half of the patients are older than 64 years at the time of diagnosis (Brodbelt et al. (2015); Thakkar et al. 2014).

Standard treatment of glioblastoma patients is, if feasible, a gross total resection of the tumour followed by fractionated tumour irradiation up to 60 Gy with concomitant systemic chemotherapy with temozolomide (TMZ) followed by six cycles of maintenance TMZ therapy (Stummer and Kamp 2009; Weller et al. 2017a). This combined chemoradiotherapy prolonged overall survival as compared to radiation therapy alone in glioblastoma patients younger than 70 years in the European Organization for Research and Treatment of Cancer (EORTC)-22,981/26,981/National Cancer Institute of Canada (NCIC) CE3 trial (Stupp et al. 2005, 2009). The benefit from combined chemoradiotherapy has been reported to decrease with increasing age of the patients (Stupp et al. 2005, 2009), a finding that might be related to an increased toxicity of the therapy in elderly patients who commonly suffer from comorbidities (Sijben et al. (2008); Tabatabai et al. 2013). In addition, differences in tumour biology, such as higher prevalence of IDH mutations in glioblastomas of younger as compared to older patients, might contribute to prognostic differences, in particular in studies that did not stratify according to IDH mutation status (Eckel-Passow et al. 2015; Hartmann et al. 2010; Houillier et al. 2010; Parsons et al. 2008). Two prospective phase III clinical trials have analysed the impact of either chemotherapy with TMZ or radiotherapy alone in the treatment of elderly glioblastoma patients (Hartmann et al. 2010; Perry et al. 2017). The NOA-08 trial compared TMZ chemotherapy (100 mg/m^2^ TMZ, one week on/1 week off protocol) with radiotherapy (30 × 1.8–2.0 Gy ad 60 Gy) in glioblastoma patients with a Karnofsky Performance Score (KPS) > 50 and an age > 65 years. Extent of resection was identified as an independent prognostic factor. Adjuvant chemotherapy was not inferior to radiotherapy but its effectiveness was related to the O6-methylguanine-DNA methyltransferase (*MGMT*) promoter methylation status (Wick et al. 2012). In elderly glioblastoma patients with *MGMT* promoter-methylated glioblastoma, TMZ chemotherapy was associated with a longer event-free survival and a trend towards longer overall survival (OS) as compared to radiation therapy alone. The opposite was true for elderly glioblastoma with *MGMT* promoter-unmethylated glioblastoma (Wick et al. 2012). In the Nordic trial (Perry et al. 2017), TMZ chemotherapy alone (200 mg/m^2^; 5/28 day cycle), radiation therapy (30 × 2 Gy ad 60 Gy) and hypofractionated radiotherapy (10 × 3.4 Gy ad 34 Gy) were compared in newly diagnosed glioblastoma patients who were older than 60 years at diagnosis. Again, chemotherapy alone was not inferior to radiation therapy alone in terms of overall survival and the *MGMT* promoter methylation status was a valuable marker for response to TMZ chemotherapy. For patients older than 70 years, OS was prolonged in the chemotherapy and hypofractionated radiotherapy groups when compared to the standard radiotherapy group (Perry et al. 2017). The CCTG CE.6/EORTC 26,062–22,061 phase III trial randomized newly diagnosed glioblastoma patients aged 65 years or older to hypofractionated radiotherapy (40 Gy/15 fractions) alone versus hypofractionated radiotherapy with concomitant and adjuvant temozolomide (Perry et al. 2017). The results of this study suggested that the addition of temozolomide to short-course radiotherapy resulted in longer survival than short-course radiotherapy alone. According to the European Association for Neuro-Oncology (EANO) guideline of 2017 (Hegi et al. 2005), treatment of glioblastoma patients should consider molecular biomarkers like IDH mutation and *MGMT* promoter methylation. Patients diagnosed with IDH-mutant glioblastoma should be treated with surgery and radiotherapy with or without concomitant TMZ followed by TMZ regardless of age. In case of IDH-wildtype glioblastoma, patients aged ≥ 70 years should be treated with surgery and hypofractionated radiotherapy in case of an *MGMT* promoter-unmethylated tumour, while elderly patients with an *MGMT* promoter-methylated tumour should be treated by surgery and TMZ plus hypofractionated radiotherapy or surgery and TMZ alone (Weller et al. 2017b). As elderly patients more often suffer from other comorbidities and higher frailty when compared to younger patients, therapeutic decisions need to be adopted to their general health status. On the other hand, elderly patients in good general condition and without any relevant comorbidities may be suitable to standard treatment according to the EOTC-22981/NCIC CE3 protocol (Stupp et al. 2005).

The aim of the present study was to analyse survival, perioperative neurologic functioning and complication rates following multimodal initial therapy in an institutional cohort of non-elderly (< 65 years) and elderly (≥ 65 years) glioblastoma patients who otherwise were in good clinical condition and were treated uniformly with open resection followed by chemoradiotherapy according to the EORTC 26,981 NCIC CE.3 protocol (Felsberg et al. 2010; Glaser et al. 2017).

## Materials and methods

### Study design, inclusion and exclusion criteria

Patients fulfilling the following inclusion criteria were considered for this study and their data were retrospectively analysed: (1) Surgical treatment at the Department of Neurosurgery, Heinrich Heine University Düsseldorf, between 01/2013 and 12/2017, (2) neuropathologically confirmed diagnosis of an IDH-wildtype glioblastoma, World Health Organization (WHO) grade IV (Louis et al. 2016), (3) primary surgical resection or surgical resection within four weeks following initial biopsy, (4) preoperative Karnofsky performance scale (KPS) ≥ 70% and (5) postsurgical therapy according to the EORTC-22981/NCIC CE3 protocol. Exclusion criteria included (1) other histopathological diagnoses than glioblastoma, IDH-wildtype (2) glioblastoma in patients with a preceding diagnosis of a WHO grade II or III glioma, (3) primary biopsy without resection within 4 weeks, (4) preoperative KPS < 70%, (5) postsurgical therapy different from the EORTC-22981/NCIC CE3 protocol (e.g., chemotherapy with TMZ and lomustine (CCNU), application of tumour-treating fields, either chemotherapy or radiation therapy alone), (6) treatment at another institution (e.g. first biopsy or surgery at another neurosurgical department).

### Surgical and postoperative treatment

All patients underwent surgical resection with standard 5-aminolevulinic acid (5-ALA) fluorescence-guided resection and intraoperative neuro-navigation. For tumours located in eloquent brain regions, surgery was planned with intraoperative monitoring and as awake surgery in an asleep–awake–asleep protocol (Kamp et al. 2012,2015b). Eloquent brain regions were defined as cortical or subcortical brain areas for which intraoperative stimulation was expected to elicit changes in neurologic condition (particularly regarding speech, movement and tactile sensation) or to elicit a response in electrophysiological recordings in corresponding areas (Kamp et al. 2015b). Extent of surgical resection was postoperatively determined by contrast-enhanced magnetic resonance imaging (MRI) within 72 h after surgery (Kamp et al. 2015a). All patients received TMZ chemoradiotherapy according to the EORTC protocol (Kocher et al. 2011) as initial treatment following resection, with at least one cycle of maintenance TMZ chemotherapy (median: six cycles, range 1–6 cycles). Radiotherapy was administered as standard fractionated therapy (30 × 2 Gy ad 60 Gy). Follow-up consisted of regular clinical and radiological re-assessment 6 weeks after end of radiotherapy and thereafter every 3 months after diagnosis.

### Histopathological and molecular analyses

All tumours were neuropathologically classified as glioblastoma, IDH-wildtype, WHO grade IV according to the WHO classification of central nervous system tumors 2016 (Louis et al. 2016). Tumours from patients diagnosed before 2016 were neuropathologically re-evaluated and reclassified according to the WHO 2016 criteria. The IDH mutation status was assessed by immunohistochemistry for IDH1-R132H as reported (Felsberg et al. 2010; Hartmann et al. 2010). Tumours of patients younger than 55 years of age were additionally investigated for less common mutations at codon 132 of *IDH1* and codon 172 of *IDH2* by Sanger sequencing or pyrosequencing as reported (Felsberg et al. 2010). The *MGMT* promoter methylation status was determined by methylation-specific PCR and pyrosequencing of sodium bisulfite-treated DNA as reported (Felsberg et al. 2009,2011).

### Study variables and neuroimaging

Preoperative Karnofsky Performance Scale (KPS), Eastern Co-operative Oncology Group Score (ECOG) and the National Institute of Health Stroke Scale (NIHSS) were determined at initial admission and postoperative KPS at discharge from the Department of Neurosurgery (Karnofsky et al. 1949) (Goldstein et al. 1989; Verger et al. 1992). In addition, KPS was assessed at follow-up visits at the department's outpatient’s office.

All MRI images were obtained by contrast-enhanced 1.5 T MRI (Avanto; Siemens, Erlangen, Germany). For detection of residual tumour tissue after surgery or for diagnosis of disease progression/tumour recurrence, non-contrast-enhanced and contrast-enhanced T1- and T2-weighted, diffusion and fluid attenuated inversion recovery sequences were evaluated. Evaluations of the MR images were performed by an attending neurosurgeon and a neuroradiologist. The extent of surgical resection was assessed by MRI within 72 h after surgery. A complete surgical resection (CR) was defined as a complete resection of the contrast-enhancing tumour tissue and was distinguished from subtotal resection (SR), in which residual contrast-enhancing tumour tissue was present in postoperative MRI. A progression/tumour recurrence was diagnosed when Response Assessment in Neuro-Oncology (RANO) criteria were fulfilled (Kamp et al. 2015a). In 81 patients (61%), additional investigations by FET-PET were performed to confirm evidence of tumour recurrence (Kamp et al. 2015b). Moreover, 77 patients were re-operated for recurrent disease and neuropathological analysis confirmed presence of recurrent tumour in 70 of these patients. 47 patients received an additional adjuvant therapy after diagnosis of tumour recurrence. Adjuvant therapy was only radiation therapy in ten patients, only chemotherapy in 21 (5/23 TMZ chemotherapy in 12 patients, weekly TMZ in seven patients, procarbazine / CCNU in two patients, only bevacizumab in one patient). One patient got only Tumour Treating Fields and 14 patients a combination of radiation and TMZ chemotherapy.

### Outcome variables

Progression-free survival was defined as the time span between initial surgery and diagnosis of tumour progression on MRI. Overall survival was defined as the time span between initial surgery and tumour-related death. Patients with unknown date of death were censored at the time of last follow-up.

### Data management

Demographic data including information on age at diagnosis and gender, KPS pre- and post-surgery as well as at 6-week follow-up following radiation, extent of resection, PFS and OS were collected retrospectively from patients’ charts. The follow-up ended on January 29, 2019 and all patients who were still alive were censored at the date of last follow-up. Continuous variables are presented as mean ± standard error of mean, ordinal values were presented as median values and minimum–maximum ranges.

All patients that fulfilled the inclusion criteria were divided into two groups, those < 65 years and those ≥ 65 years of age. The two groups were statistically compared.

### Statistical analysis

For continuous data, median and interquartile range are presented. For categorical data, frequencies and percentages are presented. Kaplan–Meier survival analysis including log-rank test and Cox regression analyses are used for statistical significance testing. Sidak’s correction was applied to adjust for multiplicity. Therefore, for statistical significance, *P* values were considered significant at a level of < 0.003. A tendency towards a correlation was defined for *p* values between 0.05 and 0.003. All statistical analyses were performed with SPSS software (Version 25.0,—IBM-, USA) and the Graph Pad Prism 5 package 3.3.2 (GraphPad Software, Inc., La Jolla, USA).

## Results

### Patient cohort

Between 2013 and 2017, 132 patients among a total of 683 glioblastoma patients (including recurrent glioblastoma) treated at our centre during this time period met the inclusion criteria for this study. The median age of the 132 patients at diagnosis was 61 (range 22–83 years). 74 patients were younger than 65 years and 58 patients were 65 years and older. In the group of the elderly patients, the median age was 70.5 years (range: 65–83 years, interquartile range, IQR: 10). Median age in the patients aged < 65 years was 55 years (range: 22–64 years, IQR: 10). In both groups, males dominated the study population: 45 (61%) in the non-elderly group and 34 (58%) in the elderly group. All patients were diagnosed with glioblastoma, IDH-wildtype, WHO grade IV according to the 2016 WHO classification of central nervous system tumours (Louis et al. 2016). *MGMT* promoter methylation was detected in 54/132 tumours (41%), including 23/74 tumours (31%) in the group of patients aged < 65y and 31/58 tumours (53%) in the elderly patient group.

### Clinical performance and adverse effects of therapy

**Fig. 1:**
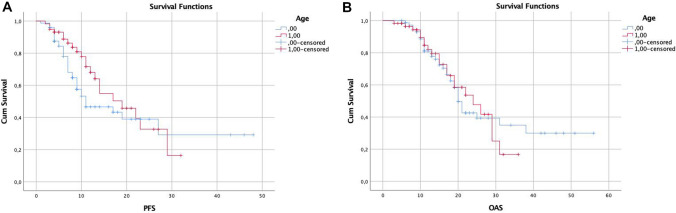
**a** Summarizes progression-free survival (PFS) and (**b**) the overall survival (OS) both stratified according to age

The median preoperative KPS was 90% for both groups (IQR: 10) and was unaffected at the day of hospital discharge after initial resection. The pre- and postoperative ECOG and NIHSS were 0 and 1 without any significant differences, respectively (each median 90, IQR: 10). While the NIHSS values 6 weeks after the end of the radiation therapy were not significantly different, KPS and ECOG values were significantly worse in the group of elderly patients at this time point. More detailed information on the neurologic performance is summarized in Table 1; Fig. 1.

**Table 1:** Differences in neurologic performance scales and adverse effects between the non-elderly and elderly patient groups over time

	*n* ( Patients < 65 years)	*n* ( Patients ≥ 65 years)	*p* value**
KPS [median (IQR)]			
Preoperative	90 (90–100)	90 (80–100)	0.17
Day of dismission	90 (90–100)	90 (82.5–97.5)	0.05
6 weeks following radiation therapy	90 (90–100)	90 (80–90)	< 0.01
After concomitant chemotherapy	90 (80–100)	80 (62.5–90)	< 0.01
ECOG (median (IQR])			
Preoperative	0 (0–0)	0 (0–1)	0.3
Day of dismission	0 (0–0)	0 (0–0.75)	0.35
6 weeks following radiation therapy	0 (0–0)	0 (0–1)	< 0.01
NIHSS (median [IQR])			
Preoperative	1 (0–2)	1 (0–2)	0.33
Day of dismission	0 (0–1)	1 (0–1)	0.14
6 weeks following radiation therapy	0 (0–2)	0 (0–2)	0.26
Adverse effects* (%)	12 (16.4)	20 (33.9)	0.02***

*KPS* Karnofsky performance score; *ECOG* Eastern Cooperative Oncology Group scale of performance; *NIHSS* National Institutes of Health Stroke Scale; *IQR* interquartile range. *Adverse effects included AZV, leukopenia, leukocytopenia, and thrombocytopenia

**p Values according to the Mann–Whitney test

***p Value according Chi Square statistic

All patients received at least one cycle of maintenance TMZ following the concomitant chemoradiotherapy. The median number of administered TMZ cycles in the entire cohort was 6 in both elderly and non-elderly patients (interquartile range 4–6, for the elderly patients: 4–6 and 6–6 for the non-elderly patients). TMZ had been stopped prematurely in 23 elderly (39%) and 18 non-elderly patients (24.7%). Reasons for prematurely stopped chemotherapy were adverse side effects. In total, 32 patients suffered from relevant adverse effects of the adjuvant therapy (23.5%). Among these, 20 patients were ≥ 65 years of age (20/58 elderly patients, 33.9%) and 12 patients were < 64 years of age (12/74 patients < 65y, 16.2%). Most commonly, patients exhibited an impairment of their general health condition as assessed by an impairment of the KPS of more than 20 points (13/58 patients ≥ 65y, 24.1%; 6/74 patients; 8.1%, *X*^2^ = 6.5, *p* = 0.01). Nine patients showed haematologic toxicities with severe thrombocyto- and/or leukocytopenia according to the common toxicity criteria grade three or four (five patients < 65 years, four patients ≥ 65 years). Two patients suffered from grade-four hepatic toxicities (CTC criteria; one patient in each age group). The incidence of haematological and hepatic toxicities did not differ significantly between age groups.

### Hazard ratios for progression-free and overall survival

A total of 56 non-elderly patients (76.7%) and 41 elderly patients (70.7%) developed tumour progression within the observation period. The median PFS was 17 months and not significantly different for both age groups (11 months vs. 19 months; *X*^2^ = 1.7; *p* = 0.2). The median OS in the present cohort was 21 months as depicted in the Kaplan–Meier survival curves. OS did not significantly differ between the two age groups (20 months vs. 24 months, Log-Rank *X*^2^ = 0.002; *p* = 0.96). Univariate hazard ratios for overall survival and progression-free survival are given in Table 2. In the univariate analyses, the degree of surgical resection and *MGMT* promotor methylation tended to correlate with the progression-free survival (for the degree of surgical resection: hazard ration, HR: 1.8; 95%-CI 1.04–3.16; *p* = 0.04; for the MGMT promotor methylation HR: 0.5; 95%-CI 0.28–0.9; *p* = 0.02) and the overall survival (for the degree of surgical resection: HR: 1.3; 95%-CI 0.75–2.28; *p* = 0.34; for the *MGMT* promotor methylation HR: 0.46; 95%-CI 0.26–0.83; *p* = 0.01). Multivariate hazard ratios revealed a tendency towards significance for the effect of the degree of surgical resection on the progression-free survival (for the degree of surgical resection: HR: 2.1; 95%-CI 1.09–4.09; *p* = 0.03). However, the *MGMT* promoter methylation was the only analysed variable that had a significant effect on overall survival. In the present cohort, age had neither an effect on the progression-free nor on the overall survival in both statistical analyses. Univariate and multivariate hazard ratios are given in (Table 2, 3).

**Table 2:** Univariate hazard ratios for overall survival and progression free survival

	OAS				PFS			
	HR	95% CI			HR	95% CI		
		Lower	Upper	*p* value		Lower	Upper	*p* value
Age								
Dichotomized	1.013	0.587	1.747	0.963	0.702	0.409	1.208	0.202
Continuous	1.003	0.98	1.026	0.805	0.99	0.969	1.011	0.351
Gender	1.32	0.771	2.258	0.312	1.552	0.635	3.797	0.335
KPS								
Preoperative	0.999	0.965	1.035	0.967	1.012	0.979	1.047	0.474
Postoperative	0.981	0.95	1.014	0.257	0.995	0.964	1.028	0.774
3rd Month of follow-up	0.975	0.95	1.002	0.067	0.981	0.954	1.008	0.173
GTR	1.308	0.751	2.277	0.343	1.808	1.036	3.156	0.037
MGMT	0.46	0.256	0.826	0.009	0.497	0.276	0.895	0.02

*OAS* overall survival; *PFS* progression free survival; *CI* confidence interval; *HR* hazard ratio; *GTR* gross total resection (0 = yes, 1 = no); *MGMT* O6-methylguanine-DNA methyltransferase (0 = unmethylated, 1 = methylated)

**Table 3:** Multivariate hazard ratios for overall survival and progression free survival

	OAS				PFS			
	HR	95% CI			HR	95% CI		
		Lower	Upper	*p* value		Lower	Upper	*p* value
Age								
Dichotomized	1.023	0.979	1.068	0.319	1.013	0.975	1.052	0.511
Continuous	0.691	0.244	1.955	0.486	0.524	0.198	1.392	0.195
Gender	1.169	0.637	2.147	0.614	1.106	0.57	2.145	0.766
KPS								
Preoperative	1.001	0.955	1.05	0.957	1.034	0.98	1.092	0.224
Postoperative	0.983	0.937	1.031	0.474	0.986	0.934	1.04	0.601
3rd Month of follow-up	0.956	0.926	0.988	0.006	0.964	0.932	0.997	0.033
GTR	1.28	0.681	2.406	0.442	2.107	1.086	4.087	0.027
MGMT	0.353	0.18	0.689	0.002	0.53	0.273	1.03	0.061

*OAS* overall survival; *PFS* progression free survival; *CI* confidence interval; *HR* hazard ratio; *GTR* gross total resection (0 = yes, 1 = no); *MGMT* O6-methylguanine-DNA methyltransferase (0 = unmethylated, 1 = methylated)

## Discussion

The present single-centre study analysed neurological performance and survival of patients diagnosed with IDH-wildtype glioblastoma and treated by surgical resection followed by concomitant and maintenance chemoradiotherapy according to the Stupp protocol (Felsberg et al. 2010; Glaser et al. 2017). A particular focus was placed on the comparison of groups of patients stratified according to age at diagnosis into elderly (≥ 65 years of age) and non-elderly (< 65 years of age) patients. There are three key findings in this study: (1) Elderly patients suffer more frequently from a deterioration of their general health condition than younger patients following initial multimodal therapy according to Stupp protocol. However, hematologic and hepatic complications of TMZ chemotherapy were similar in both groups. (2) Survival of elderly patients in good clinical condition is similar to survival of non-elderly patients. (3) *MGMT* promoter methylation was the only analyzed variable with a significant influence on OS in the multivariate analysis (Table 4).

**Table 4:** TMZ chemotherapy and adverse events

	Patients < 65 years	Patients ≥ 65 years	*p* value**
TMZ cycles (median (IQR])	6 (6–6)	6 (4–6)	0.1713
Number of patients in which TMZ had to be stopped prematurely (%)	X (x)	X (x)	
Adverse effects* (%)	12 (16.4)	20 (33.9)	
Impairment of the general health condition (%) radiation therapy	6 (8.1)	13 (24.1)	*X*^2^ = 6.5, *p* = 0.01
Haematologic toxicities (%)	5 (6.8)	4 (6.8%)	n.a
Hepatic toxicities (%)	2 (2.7)	2 (3.4)	n.a

*KPS*, Karnofsky performance score; *ECOG* Eastern Cooperative Oncology Group scale of performance; *NIHSS* National Institutes of Health Stroke Scale

*Adverse effects included AZV, leukopenia, leukocytopenia, and thrombocytopenia

**p Values according to the Mann–Whitney test

The optimal treatment of elderly patients with glioblastoma is still part of an on-going debate and has been addressed in both retrospective studies as well as prospective clinical trials (Glaser et al. 2017; Harrison and Groot 2018; Karsy et al. 2018; Morgan et al. 2017; Okada et al. 2017; Palmer et al. 2018; Pretanvil et al. 2017; Putz et al. 2016; Socha et al. 2016; Victor et al. 2019; Zhang et al. 2016). The EORTC-22981/26,981 / NCIC CE3 trial led to the establishment of concomitant TMZ chemoradiotherapy followed by TMZ maintenance therapy as standard of care for adult glioblastoma patients aged less than 70 years (Stupp et al. 2005,2009). The benefit of this multimodal therapy concept, however, decreased with increasing patient age (Sijben et al. 2008; Tabatabai et al. 2013). Two prospective randomized trials, therefore, analysed less-toxic therapy concepts based on either TMZ, standard radiotherapy or hypofractionated radiotherapy (Hartmann et al. 2010; Perry et al. 2017). Both studies found that the effectiveness of adjuvant chemo- or radiotherapy is closely linked to the *MGMT* promoter methylation status (Wick et al. 2012). Similarly, a prospective cohort study of the German Glioma Network of 233 glioblastoma patients aged ≥ 70 years revealed longer PFS (5.2 vs. 4.7 months) and OS (8.4 vs. 6.4 months) in patients with *MGMT* promoter-methylated tumours (Reifenberger et al. 2012). The NORDIC trial also reported that standard radiotherapy may be less favorable compared to chemotherapy or hypofractionated radiotherapy in patients older than 70 years (Malmstrom A, et al. 2012). In a further prospective cohort study derived from the Norwegian Cancer Registry, the median overall survival was 7.4 month in the radiotherapy alone and 13.4 month in the group treated with radiotherapy and TMZ in patients older than 70 years of age (Ronning et al. 2012). The recent CCTG CE.6/EORTC 26,062–22,061 phase III trial on glioblastoma patients aged ≥ 65 years or older similarly revealed longer survival of patients treated with hypofractionated radiotherapy and TMZ compared to hypofractionated radiotherapy alone (Perry et al. 2017).

Several neuro-oncologic centres offer elderly patients in a good general health condition adjuvant chemoradiotherapy according to the EORTC-22981/26,981/NCIC CE3 protocol (Felsberg et al. 2010; Glaser et al. 2017). Data regarding toxicity and efficacy of this approach in elderly patients with IDH-wildtype glioblastoma are still limited. The present study provides a retrospective analysis of a homogenous cohort of IDH-wildtype glioblastoma patients who were in good clinical condition at the time of diagnosis and were treated homogeneously according to the EORTC protocol. In line with the data in the original data in the EORTC-22981/26,981/NCIC CE3 trial (Stupp et al. 2005,2009), we observed a higher frequency of deterioration of the general health status in elderly patients and subsequently a higher complication rate following multi-modal initial treatment. In contrast, the frequency of haematological and hepatic complication was comparable in elderly patients and non-elderly patients of our cohort. The extent of surgical resection and the *MGMT* promoter methylation status were found to be prognostic, as documented in previous studies (Hegi et al. 2005,2008,2019; Kamp et al. 2018; Reifenberger et al. 2012; Senft et al. 2011; Stummer and Kamp 2009; Stummer et al. 2006). Interestingly, in the present study, the higher complication rate of initial therapy in elderly patients had no effect on PFS and OS, which were comparable in the groups of elderly and non-elderly patients.

The present study, thus, suggests that elderly IDH-wildtype glioblastoma patients in a good clinical condition might benefit from a similarly aggressive therapy as applied to younger patients. Although clinical deterioration following initial therapy was more common in elderly patients, PFS and OS were statistically not affected by this observation. Furthermore, our data confirm *MGMT* promoter methylation as a powerful predictor of outcome in both elderly and non-elderly glioblastoma patients. As the incidence of glioblastomas and also cerebral metastases increases with age, treatment of elderly brain tumour patients becomes increasingly more relevant in neuro-oncology and novel therapy concepts need to be developed that consider the special needs of the large geronto–neuro–oncological population of patients (Mason et al. 2016; Munoz-Bendix et al. 2019; Tabatabai et al. 2013; Wick et al. 2012; Wirsching et al. 2015).

### Limitations

We acknowledge that our present study has several limitations: (1) All data are derived from a retrospective, single-centre study. (2) By defining strict inclusion criteria (IDH-wildtype glioblastoma treated by surgical resection and standard chemoradiotherapy according to the EORTC-/NCIC-protocol, presurgical KPS > 70), we aimed to minimize potential confounders and to constitute a homogenous patient cohort. However, the analysed cohort is highly selected and, therefore, not representative of the entire patient cohort of glioblastoma patients. Furthermore, we cannot exclude potential influences related to a selection bias in this relatively small patient cohort. (3) Not all patients received the same cycles of chemotherapy due to side effects, such as hematoxicity and other reasons. We have not analysed a potential correlation between the administered amount of temozolomide and the progression-free and overall survival. (4) This cohort includes all patients who were treated in our neuro-oncologic centre between 2013 and 2017 and who met the inclusion criteria. During and after this period, neuro-oncological treatment of glioblastomas has progressed, e.g. the advent of chemotherapy with TMZ and CCNU for patients with *MGMT* promoter-methylated glioblastoma (Herrlinger et al. 2019) as well as the introduction of tumour-treating fields (Stupp et al. 2017). These concepts were not considered in the current study. “(5) In the present analysis, KPS and ECOG scores were significantly worse in elderly patients 6 weeks after the end of radiotherapy while NIHSS scores did not significantly changed. This difference might in part be related to the different metrics of the respective scales. (6) We did not perform a quality-of-life assessment. Possibly, a more aggressive therapy might lead to an impairment of the patient´s quality of life and some patients, particularly among the elderly patients, might prefer a less aggressive post-surgical therapy associated with lower life expectancy but a higher quality of life. Evaluation of the individual patient’s therapy goals by shared decision-making is additionally essential.

## Conclusion

In a retrospective analysis of 132 patients with IDH-wildtype glioblastoma treated by surgical resection and TMZ chemoradiotherapy, patients aged ≥ 65 years showed comparable PFS and OS as patients aged < 65 years. Elderly patients, however, suffered more frequently from a deterioration of their general health condition following aggressive tumour therapy than younger patients. Incidences of hematologic and hepatic toxicities of TMZ were similar in both groups. *MGMT* promoter methylation had a significant influence on PFS and OS independent from patient age.
